# Comparison of the Relationship between Lying and Standing Ultrasonography Measures of Muscle Morphology with Isometric and Dynamic Force Production Capabilities

**DOI:** 10.3390/sports5040088

**Published:** 2017-11-21

**Authors:** John P. Wagle, Kevin M. Carroll, Aaron J. Cunanan, Christopher B. Taber, Alexander Wetmore, Garett E. Bingham, Brad H. DeWeese, Kimitake Sato, Charles A. Stuart, Michael H. Stone

**Affiliations:** 1 Center of Excellence for Sport Science and Coach Education, Department of Sport, Exercise, Recreation, and Kinesiology, East Tennessee State University, Johnson City, TN 37601, USA; carrollk@etsu.edu (K.M.C.); cunanan@etsu.edu (A.J.C.); wetmore@etsu.edu (A.W.); binghamg@etsu.edu (G.E.B.); deweese@etsu.edu (B.H.D.); satok1@etsu.edu (K.S.); stonem@etsu.edu (M.H.S.); 2 Department of Physical Therapy and Human Movement Science, Sacred Heart University, Fairfield, CT 06825, USA; taberc@sacredheart.edu; 3 Department of Internal Medicine, Quillen College of Medicine, East Tennessee State University, Johnson City, TN 37601, USA; stuartc@etsu.edu

**Keywords:** ultrasonography, muscle architecture, force, strength, rate of force development

## Abstract

The purpose of the current study was (1) to examine the differences between standing and lying measures of vastus lateralis (VL), muscle thickness (MT), pennation angle (PA), and cross-sectional area (CSA) using ultrasonography; and (2) to explore the relationships between lying and standing measures with isometric and dynamic assessments of force production—specifically peak force, rate of force development (RFD), impulse, and one-repetition maximum back squat. Fourteen resistance-trained subjects (age = 26.8 ± 4.0 years, height = 181.4 ± 6.0 cm, body mass = 89.8 ± 10.7 kg, back squat to body mass ratio = 1.84 ± 0.34) agreed to participate. Lying and standing ultrasonography images of the right VL were collected following 48 hours of rest. Isometric squat assessments followed ultrasonography, and were performed on force platforms with data used to determine isometric peak force (IPF), as well as RFD and impulse at various time points. Forty-eight hours later, one-repetition maximum back squats were performed by each subject. Paired-samples t-tests revealed statistically significant differences between standing and lying measurements of MT (*p* < 0.001), PA (*p* < 0.001), and CSA (*p* ≤ 0.05), with standing values larger in all cases. Further, standing measures were correlated more strongly and abundantly to isometric and dynamic performance. These results suggest that if practitioners intend to gain insight into strength-power potential based on ultrasonography measurements, performing the measurement collection with the athlete in a standing posture may be preferred.

## 1. Introduction

Ultrasonography is commonly used to assess muscle size (e.g., muscle thickness, cross-sectional area) and architecture (e.g., pennation angle) [1,2,3], and has been shown to be valid against the gold standards magnetic resonance imaging [4,5,6] and dual energy X-ray absorptiometry [7,8]. Ultrasonography measurements are typically taken in a lying, and/or resting position, meaning that the muscle is likely evaluated in a position non-specific to upright activities. This could result in large alterations in measurements of muscle size and architecture due to the influence of gravity [9,10]. However, ultrasonography provides a level of versatility (e.g., subject positioning) that other methods do not. The adaptability of ultrasonography may be exploited to allow practitioners to develop techniques that capture muscle size and architecture in positions that maintain its functional configuration.

Muscle thickness (MT) and cross-sectional area (CSA) have previously shown moderate-to-strong relationships with magnitude of force production (*r* = 0.32–0.85) [10,11], while pennation angle (PA) has been more commonly associated with rate of force development (RFD) (*r* = 0.34–0.44) [12,13,14] when measurements are collected using ultrasonography. The non-specific nature of typical athlete positioning in ultrasonography assessment makes it plausible that the selected posture may influence the magnitude of relationship observed between muscle measurements and physical outputs. Ultrasonography techniques used to assess musculature as they relate to performance potential may be more appropriate if they closely reflect the positioning found in athletic maneuvers (e.g., standing). Standing assessments provide greater ecological validity, potentially yielding more precise associations between measures of muscle architecture and upright performance outcomes. To the authors’ knowledge, the potential influence that subject positioning may have on the relationship between muscle function and architecture has not yet been explored.

Therefore, the purpose of the current study was (1) to examine the differences between standing and lying measures of MT, PA, and CSA using ultrasonography, and (2) to explore the relationships between lying and standing measures with isometric and dynamic assessments of force production. We hypothesized that standing measurements of muscle size and architecture would have comparatively greater relationships to such measures of physical output. This may be important for practitioners that work with athletic populations, as standing ultrasonography measurements may capture the muscle in a state that more closely represents its functional configuration.

## 2. Materials and Methods

### 2.1. Muscle Size and Architecture

Fourteen resistance-trained subjects (age = 26.8 ± 4.0 years, height = 181.4 ± 6.0 cm, body mass = 89.8 ± 10.7 kg, back squat to body mass ratio = 1.84 ± 0.34) volunteered for the current investigation. Subjects were required to have spent at least the past year on a resistance-training program that involved back squats. Subjects were assessed for MT, CSA, and PA of the right vastus lateralis (VL) in both lying and standing postures using ultrasonography (LOGIQ P6, General Electric Healthcare, Wauwatosa, WI, USA) [10,15]. All subjects’ hydration status was determined using a refractometer (Atago, Tokyo, Japan) to ensure hydration status would not affect the ultrasound measurements [16]. Further, to ensure that there were minimal alterations in muscle size due to swelling, ultrasonography collection was performed at least 48 h after the most recent physical activity [17]. To determine anatomical landmark on the VL, subjects were positioned in the left lateral recumbent position with an internal knee angle of 160° ± 10°. A location half the distance between the greater trochanter and lateral epicondyle of the right femur was identified and marked. A distance 5 cm medial to the mid-femur marking was also identified and marked [9,18]. This medial marking was used for the measurement of MT. The same markings were used for both lying and standing ultrasonography measurements. All landmarks for all subjects were determined by a single practitioner, and images were collected in a repeated measures manner, and therefore any potential error would be systematic. All subjects gave informed consent, and the procedures were approved by the university’s Institutional Review Board.

### 2.2. Lying Cross-Sectional Area Measurement

Lying ultrasonography measures began with the application of a water-soluble transmission gel to the measurement site and a 16 Hz probe oriented in the short-axis, perpendicular to the VL muscle, while not depressing the skin [19]. Lying cross-sectional area (LCSA) was obtained using a panoramic image sweep in the transverse plane perpendicular to the muscle [9]. A straight-edge was placed along the skin to ensure that the probe remained along the previously established midline. Three images were obtained and saved for subsequent analysis using the software provided within the ultrasonography device [10,18].

### 2.3. Lying Muscle Thickness and Pennation Angle Measurement

The measurement site location for MT and PA measurement was the point 5 cm medial to the mid-femur mark. The ultrasonography probe was then placed in the long axis, oriented parallel to the VL muscle. The probe was held at a 90° angle to the skin surface to maintain consistent images across subjects. Consistent with CSA measurement, three images were captured and saved for subsequent analysis to determine lying muscle thickness (LMT) and lying pennation angle (LPA). Analysis was performed using the software provided within the ultrasonography device [10,18].

### 2.4. Standing Ultrasonography Measurement

**Figure 1:**
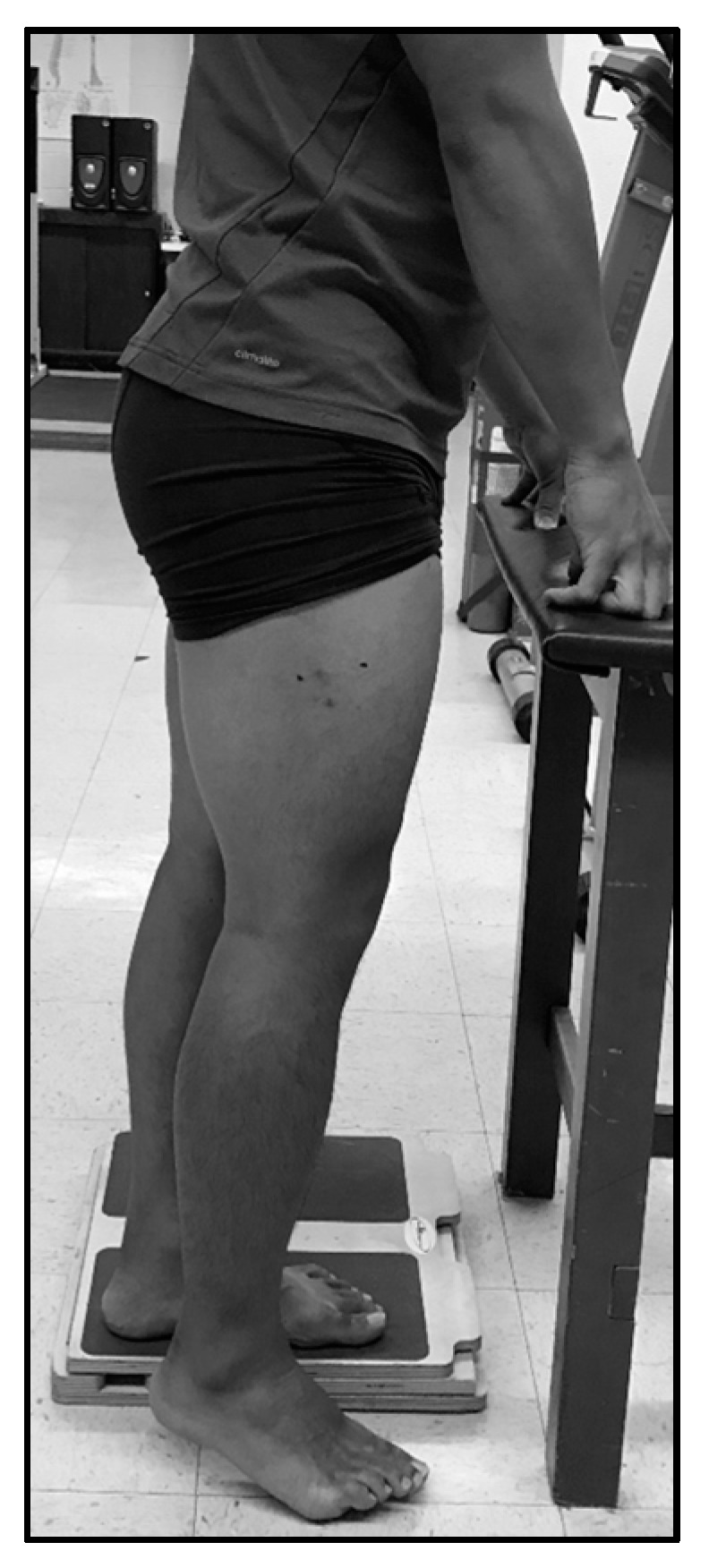
Standing ultrasonography collection position.

Following lying measures of LMT, LPA, and LCSA, standing measurements of muscle thickness (SMT), pennation angle (SPA), and cross-sectional area (SCSA) were collected. These methods were consistent with lying measures with one exception: for standing measures, the subject was upright and bearing weight on the opposite leg, which was positioned on a 5 cm tall platform, unweighting the measured leg and creating an internal knee angle of 160° ± 10° (Figure 1). Three separate long-axis images and three separate short-axis images were saved for subsequent analysis, the same as were used for the lying measurements [9].

### 2.5. Isometric Strength Assessment

**Figure 2:**
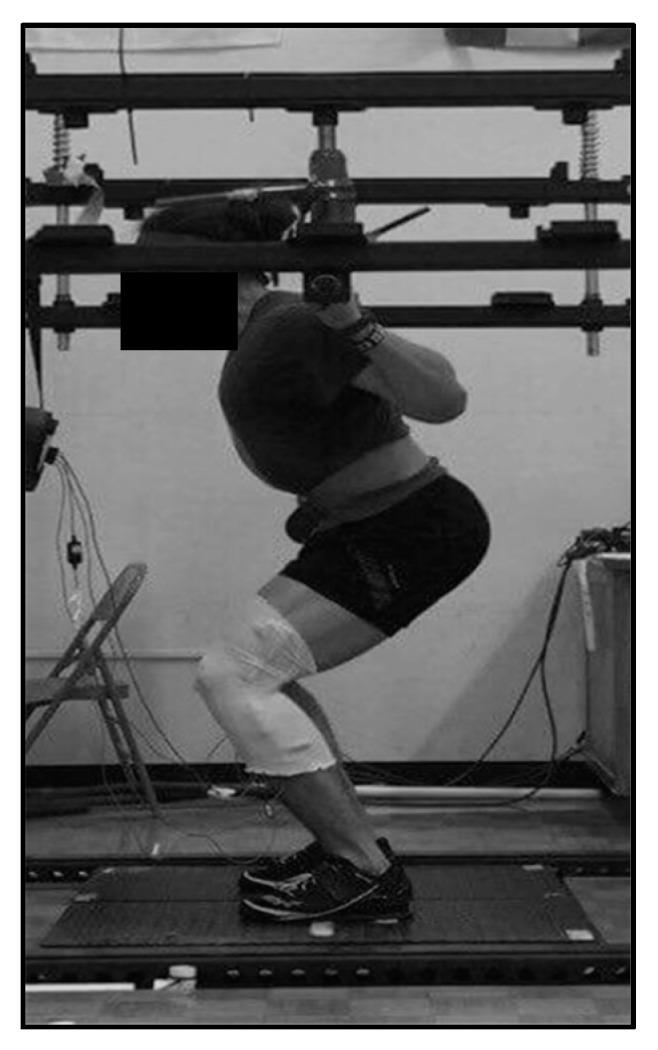
Isometric squat testing position.

Subjects completed a standardized general warm-up sequence before beginning the isometric strength assessment. After completing the dynamic warm-up, participants completed one set of five repetitions of the back squat with a 20 kg barbell followed by three sets of five repetitions at 60 kg, each separated by a 60 s rest. The isometric squat (ISQ) testing used an adapted protocol from McBride and colleagues [20,21]. Data were collected using a dual force platform design (2 × 91 cm × 45.5 cm force platforms, RoughDeck HP, Rice Lake, WI, USA) inside a custom-built apparatus, with data sampled at 1000 Hz. Participants’ bar height was set on an individual basis, to the point allowing the subject to have an internal knee angle of 100°, which was assessed using a goniometer (Figure 2) [20].

Following bar-height adjustments, participants executed ISQ trials at 50% and 75% of their perceived maximal effort. Each subject performed a minimum of two maximal effort trials. If a countermovement of greater than 200 N was observed, or trials differed by more than 250 N, subjects were required to complete an additional trial [22]. When executing maximal effort trials, subjects were first instructed to apply steady pressure on the bar before imparting maximal effort to reduce the likelihood of a countermovement. Participants were further instructed to push ‘as fast and hard as possible’ and strongly verbally encouraged during trials [20,22]. A three-minute seated rest interval was prescribed between each of the ISQ trials. LabVIEW (Version 7.1, National Instruments, Austin, TX, USA) was used for collecting and ForceDecks (Version 1.2.6464, NMP Technologies Ltd., London, UK) for processing kinetic data [23]. Isometric peak force (IPF), rate of force development over 50 ms (RFD50), 100 ms (RFD100), 200 ms (RFD200), impulse over 50 ms (IMP50), 100 ms (IMP100), and 200 ms (IMP200) were calculated from the collected data.

### 2.6. Dynamic Strength Assessment

Dynamic strength testing was conducted using a one-repetition maximum (1RM) back squat, aimed at establishing dynamic peak strength capabilities. Dynamic strength testing was completed 48 h after isometric strength assessment to allow subjects to recover from any residual effects of the previous testing [24]. Prior to testing, each subject performed a general dynamic warm-up identical to that used in ISQ testing.

Following the warm-up, the bar height and safety bar heights in the squat rack were adjusted as needed to best accommodate each subject. Subjects then performed a 1RM back squat test using a protocol modified from Suchomel and associates [25], with warm-up set intensities based on each subject’s self-reported 1RM back squat (Table 1). All subjects attempted progressively heavier loads per the protocol in Table 1 until their 1RM back squat was attained. For a repetition to be considered successful, the subject’s hip crease must have been below the patella at the bottom of the descent during the back squat, as verified by multiple certified strength and conditioning professionals.

### 2.7. Statistical Analyses

Descriptive statistics, including mean and 95% confidence interval (CI) were calculated. Normality was evaluated for each variable using the Shapiro-Wilk assessment. Within-subject reliability for each muscle morphology variable was assessed using coefficient of variation (CV) and intraclass correlation coefficients (ICC) [26]. Due to the high reliability observed for each variable (Table 2), the average of the three images was used for statistical analysis. Good reliability was also observed for all variables considered from isometric performance testing (ICC = 0.79–1.00), so the averages of two trials were used for statistical analysis. Paired-samples *t*-Tests were calculated for standing versus lying measures of the same morphological variable to determine differences between the two subject positions. Correlations between all measurements of muscle morphology and isometric and dynamic performance capabilities were calculated using Pearson’s *r*. Based on the current sample size, correlation of at least 0.53 was needed to establish a statistically significant relationship. For practical significance, Pearson’s *r* values were interpreted with magnitude thresholds previously established by Hopkins [27]. Statistical analyses were performed using JASP (Version 0.8.1.2, JASP, Amsterdam, The Netherlands) and statistical significance was set at *p* ≤ 0.05.

## 3. Results

**Figure 3:**
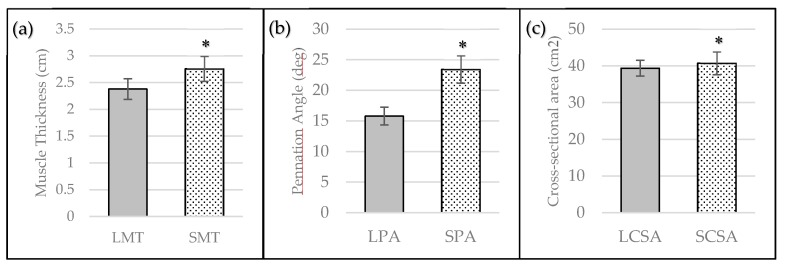
Lying and standing ultrasonography measurement differences for (**a**) Muscle Thickness; (**b**) Pennation Angle, and (**c**) Cross-Sectional Area presented as mean ± 95% CI. * = statistically significant difference compared to lying measure (*p* ≤ 0.05).

Each variable was normally distributed according to the Shapiro-Wilk assessment. Paired-samples *t*-Tests revealed statistically significant differences between standing and lying measurements of MT (*p* < 0.001), PA (*p* < 0.001), and CSA (*p* ≤ 0.05) (Figure 3). Standing measures resulted in greater values for all variables, presented as mean ± 95% CI: SMT was 14.5% ± 6.67% greater than LMT, SPA was 49.0% ± 16.0% greater than LPA, and SCSA was 3.4% ± 3.13% greater than LCSA. Additionally, standing measures related more strongly to measures of isometric and dynamic performance. The relationships between standing and lying measures of muscle morphology with isometric and dynamic performance, as well as their practical interpretation, are displayed in Table 3.

## 4. Discussion

The current investigation is the first study intended to determine the relationship between lying and standing measures of VL muscle morphology with upright isometric and dynamic performances. Although standing postures have been used in evaluating dynamic fascicle and tendon behavior [17,28], lying muscle measurements have been commonly used when the primary interest is static muscle morphology. We hypothesized that data collected using an upright posture would provide a stronger relationship to measures of standing isometric and dynamic performance. Our results indicated that (1) collection position significantly altered ultrasonography measurements of VL muscle size and architecture, and (2) standing ultrasonography measures were more strongly and more abundantly associated with measures of upright isometric and dynamic performance compared to lying ultrasonography measures.

Measures of standing muscle size (i.e., MT, CSA) and PA were statistically larger than the lying posture, providing evidence that body position substantially influenced the muscle measurements. Though a statistical change was found between the different postures with respect to CSA measures, there was a noticeably smaller percent difference compared to those of MT and PA. This indicates that while the measurements were quite different at the muscle belly, the measurements of whole muscle CSA were not influenced to the same degree. This may be due to a redistribution of the observed or neighboring muscle tissue and fluid between measurement positions due to gravity. Greater magnitude changes at the muscle belly may also be influenced by changes to fascicle orientation and/or rotation, creating a bulging effect [29]. Nonetheless, the observed increase in all measures of muscle morphology using an upright posture warrants an examination into the meaningfulness of such a difference. Most athletic actions are executed from standing postures, and therefore the potential exists that lying ultrasonography measures may not accurately capture the muscle in its functional configuration [30].

Lying measures yielded moderate correlations between LMT-1RM and LCSA-1RM, which is in agreement with previous findings [3,31,32,33]. Nevertheless, the correlations observed between standing measurements of whole muscle CSA and maximal dynamic strength were greater in magnitude, yielding a very large association between SCSA-1RM compared to a large association between LCSA-1RM. Standing CSA and SMT generated large and very large associations with IPF respectively, whereas LMT and LCSA were both considered moderate. While the relationship between muscle size as measured by ultrasonography and maximal strength has been well established [3,31,32,33], the results of the current investigation suggest that the selected posture in which muscle size is measured may influence the magnitude of its association with maximal strength. We speculate that this observation may be due to an underrepresentation of muscle size and architecture captured in a lying posture. When concerned with dynamic strength outcomes (i.e., 1RM), the relationship with MT was not considerably influenced by body position, as evidenced by both postures generating large correlations. The lack of influence position has on dynamic strength correlations could potentially be attributed to muscle-length changes during dynamic movements compared to isometric tests. Therefore, standing measures may better reflect muscle shape and architecture as they relate to upright isometric tests such as the isometric squat. It is possible that measurement of muscle architecture at a variety of joint angles may capture the changes in muscle length associated with changes in joint angle, thus better reflecting the changes in muscle length that occur during dynamic assessments. Practitioners may consider the positioning and nature of their physical assessment when determining the most appropriate ultrasonography technique in measuring muscle size.

Consideration of muscle architecture may give a more complete indication of the influence of body position on muscle imaging and the resulting associations with physical output. Pennation angle indicates fascicle orientation with respect to the aponeurosis and has been previously associated with both maximal strength and RFD [34,35]. The substantially larger SPA compared to LPA reflects the influence of gravity on muscle shape and resulting PA. Though the present investigation did not yield a significant relationship between SPA-IPF, the difference in relative magnitude of the relationships LPA-IPF and SPA-IPF should be noted. The difference in correlation coefficients further suggests that lying measures may not be accurately capturing muscle architecture as it relates to its maximal strength.

Maximal strength has been suggested to underpin RFD [36,37], as stronger athletes exhibit higher RFD and force at critical time points [35]. However, it may be valuable to assess RFD separately, as it has been found to correlate strongly with sport-specific tasks [38]. Muscle architecture is one of the major contributors to an athlete’s RFD capabilities [39,40], along with fiber-type distribution [41,42,43,44] and efferent neural drive [35,45]. In the present investigation, SPA yielded large correlations with all of the considered spectrum of RFD time points, while lying measures yielded trivial relationships. Further, large associations were observed between SMT and all RFD time points, with only small associations observed with LMT and RFD. Rate of force development during later time intervals (i.e., >100 ms) are closely related to maximal strength [36], which may also explain the observed relationship with standing measures of muscle size. The very strong correlation with SPA may be due to the greater pennation angle observed, which may be due to a more compacted arrangement of series elastic elements (e.g., actin-myosin filaments, titin, cross-bridges) [46,47,48]. The findings of the current investigation, especially considering the relationship between SPA-RFD50, suggest that standing fiber orientation may also be considered when investigating the intrinsic muscle properties influencing early-phase RFD [35,36]. Therefore, lying measures of VL muscle architecture may misrepresent the functional configuration and RFD potential entirely, limiting ultrasonography’s usefulness as a monitoring tool for strength-power athletes. Because of RFD’s implication for sporting success [35], practitioners should instead consider standing measures of muscle architecture.

Impulse combines elements of magnitude and rate of force production, as increases in either would result in an increase in impulse. Impulse has well-established relationships to sprint [49,50,51] and change-of-direction performance [52], making it potentially the most important kinetic characteristic to consider in evaluating the overall success and potential transfer of effects resulting from a training intervention. Within the current investigation, the results suggest that standing ultrasonography measures may provide a more useful representation of VL architecture in predicting impulse potential across a range of time points. All impulse variables considered (IMP50, IMP100, IMP200) elicited statistically large associations with SMT and SCSA, but no statistical significance was reached with any lying measures of muscle size. Additionally, SPA returned substantially larger correlation magnitudes compared to LPA, further suggesting that standing measurements more accurately capture the functional configuration of VL architecture as it relates to the physical potential of strength-power athletes.

## 5. Conclusions

The results of the current investigation demonstrated that ultrasonography measurements of VL muscle size and architecture were significantly larger during standing ultrasonography imaging. This is valuable considering the desire for practitioners to capture the muscle in a state that more precisely represents its configuration during performance. Further, standing ultrasonography measures were overall more strongly associated with measures of isometric and dynamic performance. This suggests that, if practitioners intend to gain insight into strength-power potential based on ultrasonography measurements, performing collection with the athlete in a standing posture is preferred.

## Figures and Tables

**Table 1:** Back squat warm-up.

Sets × Repetitions × Intensity (% 1RM)	Rest Interval
1% × 5% × 30%	1 min
1% × 3% × 50%	1 min
1% × 2% × 70%	2 min
1% × 1% × 80%	3 min
1% × 1% × 90%	3 min
1RM attempts	3 min

**Table 2:** Reliability for each muscle size and architecture variable in lying and standing postures.

Measure	CV	ICC
LMT	2.03%	0.98
SMT	1.40%	0.99
LPA	6.65%	0.90
SPA	6.18%	0.84
LCSA	1.93%	0.95
SCSA	3.63%	0.91

CV = coefficient of variation; ICC = intraclass correlation coefficient; LMT = lying muscle thickness; SMT = standing muscle thickness; LPA = lying pennation angle; SPA = standing pennation angle; LCSA = lying cross-sectional area; SCSA = standing cross-sectional area.

**Table 3:** Relationships between muscle size and architecture with measures of isometric and dynamic performance.

Measure	Outcome	IPF	RFD50	RFD100	RFD200	IMP50	IMP100	IMP200	1RM
LMT	Pearson’s *r*	0.46	0.29	0.27	0.18	0.32	0.33	0.32	0.56 *
	*p*-value	0.10	0.31	0.35	0.55	0.26	0.25	0.26	0.04
	Interpretation	Moderate	Small	Small	Small	Moderate	Moderate	Moderate	Large
SMT	Pearson’s *r*	0.73 *	0.59 *	0.53 *	0.52	0.54 *	0.58 *	0.59 *	0.55 *
	*p*-value	<0.01	0.03	0.05	0.06	0.04	0.03	0.03	0.04
	Interpretation	Very Large	Large	Large	Large	Large	Large	Large	Large
LPA	Pearson’s *r*	0.20	−0.04	0.02	−0.03	0.13	0.11	0.09	0.46
	*p*-value	0.49	0.90	0.95	0.91	0.67	0.72	0.76	0.10
	Interpretation	Small	Trivial	Trivial	Trivial	Small	Small	Trivial	Moderate
SPA	Pearson’s *r*	0.49	0.59 *	0.66 *	0.54 *	0.38	0.47	0.53 *	0.32
	*p*-value	0.08	0.03	0.01	0.05	0.18	0.09	0.05	0.26
	Interpretation	Moderate	Large	Large	Large	Moderate	Moderate	Large	Moderate
LCSA	Pearson’s *r*	0.38	0.33	0.25	0.27	0.52	0.49	0.44	0.60 *
	*p*-value	0.18	0.25	0.38	0.36	0.06	0.08	0.11	0.03
	Interpretation	Moderate	Moderate	Small	Small	Large	Moderate	Moderate	Large
SCSA	Pearson’s *r*	0.58 *	0.50	0.48	0.46	0.62 *	0.63 *	0.61 *	0.77 *
	*p*-value	0.03	0.07	0.08	0.10	0.02	0.02	0.02	<0.01
	Interpretation	Large	Large	Moderate	Moderate	Large	Large	Large	Very Large

* = statistically significant relationship (*p* ≤ 0.05). LMT = lying muscle thickness; SMT = standing muscle thickness; LPA = lying pennation angle; SPA = standing pennation angle; LCSA = lying cross-sectional area; SCSA = standing cross-sectional area; IPF = isometric peak force; RFD50 = rate of force development at 50 ms; RFD100 = rate of force development at 100 ms; RFD150 = rate of force development at 150 ms; RFD200 = rate of force development at 200 ms; IMP50 = impulse at 50 ms; IMP100 = impulse at 100 ms; IMP150 = impulse at 150 ms; IMP200 = impulse at 200 ms; 1RM = one-repetition maximum back squat.
